# *Trichinella* infections in animals and humans of Iran and Turkey

**DOI:** 10.3389/fmed.2023.1088507

**Published:** 2023-02-02

**Authors:** Mehdi Borhani, Saeid Fathi, Majid Fasihi Harandi, Sami Simsek, Haroon Ahmed, Xiaoxia Wu, Mingyuan Liu

**Affiliations:** ^1^State Key Laboratory for Zoonotic Diseases, Key Laboratory of Zoonosis Research, Ministry of Education, Institute of Zoonosis, College of Veterinary Medicine, Jilin University, Changchun, China; ^2^Department of Parasite Vaccine Research and Production, Razi Vaccine and Serum Research Institute, Karaj, Iran; ^3^Research Center for Hydatid Disease in Iran, Kerman University of Medical Sciences, Kerman, Iran; ^4^Department of Parasitology, Faculty of Veterinary Medicine, University of Firat, Elaziğ, Türkiye; ^5^Department of Biosciences, COMSATS University Islamabad (CUI), Islamabad, Pakistan

**Keywords:** trichinellosis, *Trichinella*, epidemiology, Turkey, Iran

## Abstract

Trichinellosis is considered as a cosmopolitan zoonosis caused by different species of the small nematodes of the genus *Trichinella*. The present study aimed to provide a broad review for exploring *Trichinella* sp. infection in humans and animals of Iran and Turkey. Additionally, we aimed to explore bases for trichinellosis prevention and control. Two reports of human trichinellosis following the consumption of meat of wild boar are available in the northern Iran. A large outbreak of trichinellosis and some other sporadic cases are reported mainly as a result of eating wild boar or pork meat from Turkey, where *T. britovi* is present. Field studies show that *Trichinella* sp. infections occur in wild carnivores of Iran, particularly the golden jackal (*Canis aureus*) as the most frequently infected species. *T. britovi* has been reported to be present elsewhere in Iran in wild mammals, where wild boar is the main source of *Trichinella* sp. infection. In Turkey, *Trichinella* spp. has been reported from animals including both domesticated and wild pigs and gray wolf (*Canis lupus*). However, current data on the distribution of *Trichinella* taxa are fragmentary in the Anatolian region.

## Introduction

Trichinellosis (formerly trichinosis) is a zoonotic disease caused by the consumption of meat and meat derived products infected by nematode larvae of the genus *Trichinella*. This disease represents an important health burden for humans (1). The control of disease in domesticates susceptible animals (e.g., pigs and horses) and game (mainly mammals) implies further economic burdens (2). Carnivores and domesticated and wild swine are the most important natural reservoir hosts of these nematodes (3).

From the 19th century onward, the sylvatic cycle of *Trichinella* spp. has been documented in all the continents with the exception of Antarctica (4). In the last 20 years, epidemiological data of *Trichinella* spp. infections in animals/and or human are reported from 95 (48.5%) countries worldwide including the wild cycle (in 75 countries; 38.3%), the domestic cycle (in 32 countries; 16.3%), and human infections (in 47 countries; 23.9%). However, epidemiological information is still lacking in many of countries or dates back to the last century (4).

An overall annual trichinellosis incidence rate of 469.2–985.3 cases per billion persons per year, and a global mortality rate of 0.3 to 0.8 per billion persons per year, have been estimate for trichinellosis with a global disability-adjusted life years (DALY) of 523 in 2010 (5). Most *Trichinella* sp. infections occur in the wildlife, the spillover of these parasites from wildlife to domesticated animals represents a challenge for disease control in endemic areas. There were rare reports of human trichinellosis in Iran and Turkey. *Trichinella* sp. has been reported from wild life (carnivores and wild boars) of the Caspian region, Isfahan, Ardabil, Khuzestan, Khorasan Razavi, and Bandar Abbas in the north, central, northwest, southwest, north-east and of south of Iran. *T. britovi* is circulated among wild life of Iran (6–9). A large outbreak of trichinellosis and some other sporadic cases are recorded from Turkey, where *T. britovi* was identified in meat balls and human biopsies as well as gray wolf (10, 11). *Trichinella* infection had previously been documented in both domestic and wild pigs (12). The two neighboring countries of Turkey, and Iran have historically shared socioeconomic, linguistic, and ethnic traits.

**FIGURE 1:**
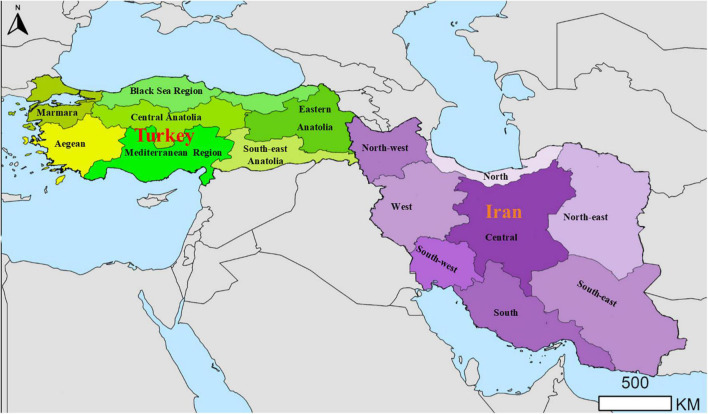
Geographical districts of Iran and Turkey.

The aim of this review was to describe the epidemiological picture of *Trichinella* spp. infections in animals and humans of Iran and Turkey (Figure 1). In-depth understanding of the epidemiology of trichinellosis can be valuable for developing control programs in a cost-effective way in order to reduce this transmission of the parasite.

## Human trichinellosis in Iran

Since the vast majority of the population of Iran and Turkey are Muslim, pig farming and consuming pork meat is rare in Muslims countries because of Islamic law. A human infection has been reported in 1966 in the northern Iran based on the clinical symptoms, seropositivity and a history of wild boar meat consumption (13). Almost, half a century later, an outbreak was documented by serology in two household members of a Tehrani family following the consumption of meat of a wild boar hunted in the Javaher–Dasht forest of Siahkal, Gilan province in 2007 (14). Human trichinellosis has not yet been identified in Iran; however, based on the sylvatic cycle, it is likely that *T. britovi* could be the causative agent of the human infection in the region (6, 15).

In Mazandaran province, anti-*Trichinella* IgG were reported in eight males out of the 364 at high-risk persons (2.2%; CI 95%, 1.9–2.4) who had consumed wild boar meat at least once in a year. The seropositivity was linked to occupation (hunters; OR, 13.5; 95% CI, 3.1–59.4) and frequency of wild boar meat consumption (more than 7 times; OR, 17.5; 95% CI, 3.2–93.6), indicating great exposer of male hunters and their friends and relatives to infected meat. Seropositivity was found to increase by age and to peak in 41–60-year-olds, but not at a significant level (16).

A study detected anti-*Trichinella* IgG by ELISA in five (2.6%) out of 189 persons suspected to be wild boar meat consumers of the Golestan Province, northern Iran (17). Serum samples of 24 persons of Bojnord, the capital city of northern Khorasan, tested negative for anti-*Trichinella* IgG by ELISA, while a serum sample of a person of 16 tested of Behshahr, a town of the Mazandaran province, tested positive (17). Three out of five seropositive persons were ≥50 year old and four out of five were males (17).

## *Trichinella* spp. infection in animals of Iran

**FIGURE 2:**
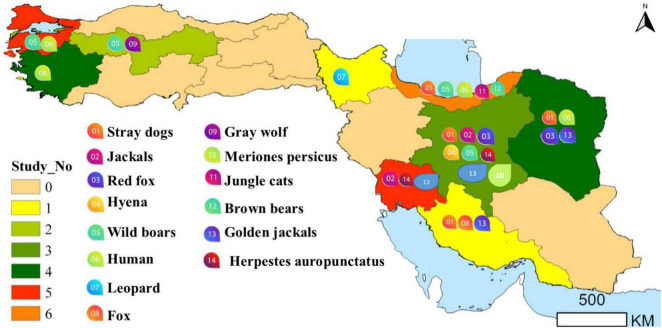
The region of the detection of *Trichinella* infected human and animals (for more details, please refer to the main text).

Until the 1990s, *Trichinella spiralis* was considered the only species in the genus and only in the last 30 years the multispecies concept has been affirmed (4). It follows that *T. spiralis* has been reported in animals from all continents in the past (Figure 2).

### Wild swine

As shown in Table 1, there are epidemiological studies on *Trichinella* sp. using different techniques. In Iran, this species was reported in two out of 4,950 carcasses of wild boar hunted in Mazandaran and Guilan provinces from 1961 to 1967 (18). Mobedi et al. (19) reported a low prevalence in wild boar (0.02%) and brown bears (6.25%) of the Caspian region hunted from 1967 to 1971. In 1978, *Trichinella* sp. larvae have been collected from wild boar (25.0%) and brown bears (6.2%) (20).

**TABLE 1:** Epidemiological investigations on *Trichinella* sp. in human and animals of Iran.

Year of study/Region	Detection tests	Hosts (positive/tested)	References
1961–1967/NORTHERN AND CENTRAL	Trichinoscopy and artificial digestion	4,950 carcasses of wild boars (2) and 21 dogs (0)	(18)
1967–1971/CASPIAN REGION	Trichinoscopy and artificial digestion	21,143 Wild boars (5),16 Brown bears (1), 63 Golden jackals (38), 3 Jungle cats (2), 3 Other carnivores (0), 30 Rodents (0), 20 Shrews (0)	(19)
1973/CENTRAL	Trichinoscopy and artificial digestion	10 Stray dogs (2), 18 Jackals (10), 18 Red foxes (2), 1 Hyena (1), 43 *Mus musculus* (0), 9 *Cricetulus migratorius* (0), 29 *Meriones persicus* (1), 15 *Nesokia Indica* (0), 13 *Meriones crassus* (0), 2 *Apodemus sylvaticus* (0), 11 *Rhombomys opimus* (0)	(23)
1969–1976/NORTHEASTERN	Trichinoscopy and artificial digestion	16 Golden jackals (8), 10 Red fox (3), 13 Hedgehog (0), 13 Shrews (0), 7 Hares (0), 21 Pika (0), 56 *Asiatica jerboa* (0), 4 Dormice (0), 160 House mice (0), 206 Wood mice (0), 10 Indian scaly tailed murine rat (0), 7 Rat (0), 108 Hamster (0), 69 Voles (0), 126 Gerbil (0)	(24)
1969–1976/SOUTH	Trichinoscopy and artificial digestion	17 Fox (4), 12 Stray dog (4), 3 Golden jackals (3), 140 *Gerbillus nanus* (0), 33 *Apodemus demitiatus* (0), 13 *Tatera indica* (0), 8 *Nesokia indica* (0), 3 *Mus musculus* (0), 2 *Rattus rattus* (0), 2 *Calomyscus bailwardi* (0), 2 *Meriones lybicus* (0), 2 *Meriones persicus* (0)	(25)
1987/NORTH AND SOUTH-WEST	Trichinoscopy and artificial digestion	1 Jackal (1), 1 *Canis aureus* (1)	(27)
2007/NORTHERN	Artificial digestion of muscle, and molecular methods	1 Wild boar (1)	(21)
2014–2017/NORTHERN	Artificial digestion of muscles, serological and molecular methods	79 Wild boars (3), 364 Human at-risk individuals (8)	(16)
2009–2010/SOUTH-WEST	Artificial digestion of muscles, and molecular methods	14 Dogs (0), 18 Jackals (2)	(7)
2013/SOUTHWESTERN	digestion of muscle	25 Wild boars (0)	(43)
2009/NORTH WESTERN	Trichinoscopy, artificial digestion of muscle, and molecular methods	1 Leopard (1)	(9)
2017/SOUTHWESTERN	digestion of muscle	52 Rodents (0)	(70)
2012–2014/NORTHERN	digestion of muscle	21 Wild boars (0)	(71)
2016–2017/NORTHEASTERN	Trichinoscopy, digestion of muscle, and molecular methods	295 Stray dogs (5), 1 Red fox (0), 12 Golden jackals (1), 1 Wild boar (0)	(8)
2010–2011/NORTHEAST	digestion of muscle, Histopathological examination and molecular methods	120 Stray dogs (3), 26 Wild boars (0), 25 Rodents (0), 2 Foxes (0), 2 Hyena (0)	(6)
2015/NORTHERN	digestion of muscle, and molecular methods	35 Wild boars (2)	(15)
2020/NORTHEAST	Serology	364 Human individuals (5)	(17)
2000/SOUTHWESTERN	digestion of muscle	10 *Herpestes auropunctatus* (3)	(31)

In 2006, *Trichinella* sp. larvae were found in two of 60 wild boars of the Khuzestan province (20).

*Trichinella* sp. larvae collected from a wild boar of the Gilan province (northern Iran), were erroneously identified as belonging to the *Trichinella murrelli*, a species of North America (21). Subsequently, a more in-depth molecular study showed that the larvae belonged to *T. britovi* (22).

No *Trichinella* sp. larvae were detected in 26 wild boars of Khorasan Razavi Province (northeast of Iran) by artificial digestion (6).

*Trichinella britovi* larvae were detected in two (5.7%) of the 35 wild boars in Amol County from 2015 to 2016 (15) and in three (3.7%) of 79 wild boars of Mazandaran Province, northern Iran during 2014–2015 (16). The five animals harbored from 0.05 to 9 larvae per g of muscle tissue [Rostami et al. (15, 16)].

### Carnivores *(jackal, fox, wolf, dog, felids, mongooses, badger)*

As summarized in Table 1, different studies have reported *Trichinella* spp. in carnivores. In 1972, *Trichinella* sp. larvae were detected in 38 (60.3%) of 63 golden jackals (*Canis aureus*) and two of three jungle cats (*Felis chaus*) of the Nour forest near Amol and Hashtpar, Caspian region, northern Iran (23). In 1973, *Trichinella* sp. larvae have been detected in two stray dogs, ten jackals, two red foxes (*Vulpes vulpes*), and one hyena (*Hyaena hyaena*) of Isfahan by Sadighian et al. (23). In 1967 and 1976, *Trichinella* sp. larvae was detected by trichinoscopy and artificial digestion of muscles in 8 (50%) out of 16 golden jackals and in 3 (30%) out of 10 red foxes collected in the eastern part of the Caspian region and northeastern Iran including Gilan, Mazandaran, Golestan, North Khorasan and Khorasan Razavi provinces (24). From 1967 to 1971, a study in the Caspian region, did not detect any *Trichinella* sp. larva in badger (*Meles meles*) (19).

Using trichinoscopy, Hamidi and Mobedi (25) detected *Trichinella* sp. larvae, identified as *T. spiralis* at that time, in 4 out of 17 red foxes, 4 out of 12 stray dogs and in three golden jackals of the Bandar Abbas region, Hormozgan Province, southern Iran.

In 1978, *Trichinella* sp. larvae have been detected in 55.7% of golden jackals (55.7%), red foxes (8.3%), hyenas (2 of 2), and stray dogs (4.2%) (26). In 1987, *Trichinella* sp. larvae from golden jackals of the Caspian Sea and Khuzestan regions, southwest of the country were identified at the species level as *T. spiralis* and *Trichinella nelsoni* (27), today *T. britovi* (28) by cross-breeding experiments in laboratory mice (29). The Khuzestan isolate (formerly *T. nelsoni*) showed a low infectivity to albino rats, whereas the Caspian isolate was found to be highly infective for albino rats and wild pigs (27) suggesting that it was a true *T. spiralis* isolate. In 2000, *Trichinella* sp. larvae (*T. spiralis* at that time) were detected in one (1.4%) of 75 stray dogs of Isfahan (30).

In 2000, *Trichinella* sp. larvae were detected in three of ten mongooses (*Herpestes auropunctatus*), of the Khuzestan province, southwest Iran. These *Trichinella* sp. larvae (named *T. nelsoni* at that time) showed a higher larval burden in laboratory mice than in laboratory rats, suggesting to be *T. britovi* (31). In 2006 in the same province, *Trichinella* sp. larvae were detected in two out of ten mongooses and in a wild cat (20).

Mowlavi et al. (9) identified *T. britovi* in a leopard (*Panthera pardus saxicolor*) hunted in Germi County, Ardabil Province, north-western Iran.

A study by Borji et al. (6) documented by artificial digestion, *Trichinella* sp. larvae, identified as *T. britovi* by multiplex PCR, in three (2.5%) of 120 stray dogs of Khorasan Razavi Province, north-east Iran.

During the period 2009–2010, Mirjalali et al. (7) investigated muscle samples of 14 stray dogs and 18 golden jackals of the Khuzestan Province, south-west Iran, where *T. britovi* had been detected 30 years earlier. The circulation of *T. britovi* was confirmed in two golden jackals [Mirjalali et al. (7)]. Punctiform deletions or substitutions in the 5S and ITS2 sequences were detected in these two *T. britovi* isolates (22).

Shamsian et al. (8) investigated 295 stray dogs, 12 golden jackals, one red fox and one wild boar of the Khorasan Razavi Province, north-eastern Iran, in the period 2016–2017. *T. britovi* larvae were detected in 5 (1.7%) stray dogs from Mashhad city and in one (8.3%) golden jackal from the surroundings of Sabzevar city.

### Insectivores, rodents and mall mole-like mammals

From 1967 to 1971, a study in the Caspian region, did not detect any *Trichinella* spp. larvae in rodents (house mouse, *Mus musculus*; wood mouse, *Apodemus sylvaticus*; black rat, *Rattus rattus* and shrews (*Crocidura* sp.) (19). An old study reported the presence of *T. spiralis* in a Persian jird (*Meriones persicus*) of Isfahan in central part of Iran (23).

From 1967 to 1976, no *Trichinella* spp. larvae were detected by trichinoscopy, and artificial digestion, in different small mammals including 13 hedgehogs (*Erinaceus europaeus*), 13 shrews (*Crocidura lucodon*); 28 lagomorpha: 7 hares (*Lepus capensis*) and 21 pika (*Ochotona rufescens*); and 746 rodents: 56 asiatica jerboas (*Allactaga elater*), 4 dormice (*Gils glis*), 160 house mice (*M. musculus*), 206 wood mice (*A. sylvaticus*), 10 Indian scaly tailed murine rats (*Nesokia indica*), 7 rats (*Rattus ratoides*), 108 hamsters (*Cricetulus migratorius* and *Calomyscus balewadi*), 69 voles (*Microtus transcapicus*, *M. Socialis, M. arvalis*, and *M. nivalis*) and 126 gerbils *(Meriones persicus, M. Crassus*, and *Rhombomys opimus*) collected from Gilan, Mazandaran, Golestan, North Khorasan and Khorasan Razavi provinces, where *Trichinella* sp. larvae had been detected in golden jackals and red foxes (24). A study by Hamidi and Mobedi (25) investigated 204 rodents using trichinoscopy and artificial digestion from the Bandar Abbas area of Hormozgan province, southern Iran, but no *Trichinella* spp. larvae were found.

Borji et al. (6) in Khorasan Razavi province, where *T. britovi* has been detected in stray dogs, no *Trichinella* sp. larvae were found in 25 rodents (species unknown).

## Human trichinellosis in Turkey

In Turkey, pork intake is uncommon, as most of population are Muslim, and consequently, trichinellosis is thought to be extremely rare. The epidemiological studies and detected regions of human trichinellosis in Turkey have been summarized in Table 2 and Figure 2.

**TABLE 2:** Investigations on *Trichinella* sp. in human and animals of Turkey.

Year of study/Region	Technique used	Sample size (positive)	References
2005/AEGEAN REGION	Serology	76 Human (47; 62%)	(34)
2021/INNER ANATOLIAN REGION	Multiplex PCR and DNA sequencing	1 Gray wolf (1)	(10)
2006/AEGEAN REGION	Serology	474 Human (154; 32.5%)	(35)
1987/MARMARA REGION	Trichinoscopy and the digestion method	535 Pigs (0), 1165 Wild boars (2)	(72)
2009/AEGEAN REGION	Trichinoscopy	43 Wild boars (0)	(40)
2011/MARMARA REGION	Trichinoscopy and artificial digestion	27 Wild boars (0), 22 Wild boars (0)	(37)
1999/MARMARA REGION	Trichinoscopy and artificial digestion	1 Wild boar (1)	(36)
2021/MARMARA REGION	Trichinoscopy and artificial digestion	59 Wild birds (0)	(39)
1977/MARMARA REGION	Trichinoscopy and artificial digestion	13 Human (13)	(32)
1971/INNER ANATOLIAN REGION	Trichinoscopy and artificial digestion	70 Wild boars (1)	(38)

In 1977, an outbreak of 13 human trichinellosis cases was documented in Kumkapi (Istanbul) following the consumption of pork (32). At that time, the etiological agent was considered to be *T. spiralis*. In 2003, an outbreak (7 infected persons) due to the consumption of wild boar meat occurred in Bursa (33).

Between December 2003 and January 2004, a large outbreak of trichinellosis (418 infected persons) occurred in Izmir, Western Anatolia. Raw minced meat balls illegally made with both beef and pork were the source of the outbreak. This food traditionally made with uncooked veal and named *cig kofte*, was very popular in Izmir. Infected raw balls were made by a wholesale meat butcher and consumed in 14 restaurants and street vendors. *Trichinella* sp. larvae collected from these meat balls and human biopsies were identified as *T. britovi* [Akkoc et al. (11)]. Information on this outbreak is also available in other papers [Ozdemir et al. (34); Turk et al. (35)], which investigated smaller groups of patients all included in the Akkoc et al. (11) study.

In 1997, *Trichinella* sp. larvae, considered to belong to *T. spiralis*, were detected in a wild boar sausage of Bulgarian immigrants in Istanbul (36).

## *Trichinella* sp. infections in animals of Turkey

### Wild boar and pigs

Wild boar is widespread across the country, however, there is no accurate estimate of their population. This game is shot by hunters, ethnic groups, or farmers for meat, and/or crop protection (37). Trichinellosis had been documented in both domesticated and wild pigs (12). From 1971 to 2011, the prevalence of *Trichinella* sp. in wild boar and domesticated pigs was investigated by six studies carried out in Istanbul, Aegean Region, Aydin, and Bursa (Table 2).

In 1971, one wild boar out of 70 tested positive in Ankara for *Trichinella* sp. larvae by trichinoscopy and artificial digestion (38). Muscle samples of 535 pigs and 1,165 wild boars were tested in Istanbul between 1973 and 1984. Two wild boars tested positive by trichinoscopy and artificial digestion (12). In 1991, 45 wild boars were examined in Aegean Region by trichinoscopy (39). In 2009, muscle samples of 43 wild boars of the Aydin province were tested by trichinoscopy, but no larvae were detected (40).

In the Bursa province, 27 wild boars were necropsied to investigate the helminth fauna including *Trichinella* sp. larvae using trichinoscopy and artificial digestion. All muscle samples tested negative (37).

### Carnivore mammals and birds

*Trichinella* sp. larvae collected from muscle samples of a gray wolf (*Canis lupus*) of Çankiri, were identified at the species level as *T. britovi* (10).

In 2021, muscle samples of from 59 different wild birds including: the common buzzard (*Buteo buteo*), house sparrow (*Passer domesticus*), yellow-legged gull (*Larus michahellis*), white stork (*Ciconia ciconia*), common magpie (*Pica pica*), barn owl (*Tyto alba*), Jackdaw (*Corvus monedula*), common swift (*Apus apus*), common kestrel (*Falco tinnunculus*), Eleonora’s falcon (*Falco eleonorae*), short-toed snake eagle (*Circaetus gallicus*), alpine swift (*Apus melba*), Eurasian hoopoe (*Upupa epops*), carrion crow (*Corvus corone*), European nightjar (*Caprimulgus europaeus*), common pigeon (*Columba livia*), tree pipit (*Anthus trivialis*) and hooded crow (*Corvus cornix*) of the Bursa province were tested for *Trichinella* sp. larvae by trichinoscopy and artificial digestion. No *Trichinella* sp. larvae were detected (39). The detected regions of animal trichinellosis in Turkey have been summarized in Figure 2.

## Epidemiological factors of trichinellosis in Iran and Turkey

It is worth noting that the decrease in backyard and free-range pig farming, the systematic regulation of abattoir facilities, and the exponential increase in pigs reared in controlled situations are among the reasons for the significant reduction of *trichinellosis* in industrialized countries (41). Despite the reduction of the domestic cycle of *Trichinella* sp., trichinellosis can still be considered as a potential risk, due to the presence of these nematodes in the wildlife, which can be a spillover for domesticated susceptible animals (4).

The low seropositivity rate or near absence of human cases in Iran is mainly due to the religious ban, which prohibits the consumption of meat from domesticated and wild swine. The wild boar is the source of *Trichinella* sp. infection for religious minorities in Iran practicing wild boar hunting (16, 42, 43).

Raw meatballs made from minced meat, wheat, tomato paste and spicy spices is traditionally made with venison or beef, while its replacement with infected pork has led to a trichinellosis outbreak in Izmir. Such outbreaks indicate that pork or wild boar meat consumption in areas without control may lead to serious health and economic problems, even when religion limits consumption (44). Thus, the presence of trichinellosis in Muslim societies, as occurred in Turkey, reveals that these societies are also at risk of trichinellosis.

*Trichinella britovi* was confirmed to be causative agent of infection in Turkey (11, 34). *T. britovi* is mostly a sylvatic species, although it can infect humans when farmers fail to properly rear domestic pigs, allowing them to come into touch with animals. A hunted wild boar or a free-range or backyard pig could be the source of infection (11). These outbreaks highlight the importance of testing swine for *Trichinella* infection at the slaughterhouse to ensure safe food for consumers, particularly when raw meat is consumed. Educational strategies such as health communication campaign for consumers, farmers, hunters and other groups can be of great importance. In these outbreaks, early detection, notification of health authorities, and identification of affected meat had led to the control of infection (35).

The most key routes of transmission of *Trichinella* taxa are predation and scavenging behavior. At present, only *T. britovi* has been detected in wild animals of Iran and Turkey. More sampling is needed to reveal the spread of this species in these two countries of Western Asia. In Iran, current data indicates the golden jackal as an indicator animal for the circulation of *T. britovi* because of its scavenging behavior and adaptability to various habitats, including the peri-domestic habitats (8, 45, 46).

### Trichinellosis prevention and control in human and animals

Regarding the lack of pig farming and the rare human cases, only public and professional education, and preventing illegal wildlife hunting can be recommended for prevention and control of disease in the region.

### Health communication and One Health approach (OH)

As mentioned high risk behavior (i.e., hunter-based life styles and recreational hunting leaving animal carcasses in filed, discarding game carcasses together with household garbage) can favor *Trichinella* sp. transmission in the wild (4). Educational plans (such as Health communication campaign) are in need of the commitment, coordinated actions under OH approaches to increase awareness of consumers, farmers and hunters, and compliance regarding trichinellosis especially in rural communities and remote areas (e.g., high-risk regions), where comprehensive data are needed on the circulation of *Trichinella* sp. in wildlife. The One Health triad is made up of humans, domestic animals, wildlife and the changing ecosystems. Human activities affect the flow of all parasitic infections in the One Health triad, and the impact of the resulting spillover events (e.g., spillover from wildlife to humans) are considered (47).

The importance of the One Health approach in the control of infectious diseases should be well embraced by the relevant authorities for providing public education on zoonotic diseases using media, radio, television, newspapers (48) science exhibitions, media production and other tools, where crucial changes of care policy and educational system will be needed to increase the knowledge of the public, particularly in the low- and middle-income settings (49).

It has been indicated that communication campaigns over longer time periods can be capable of changing health behaviors and increasing awareness of public regarding pork-borne parasitic diseases and schistosomiasis control measures (50–53).

**FIGURE 3:**
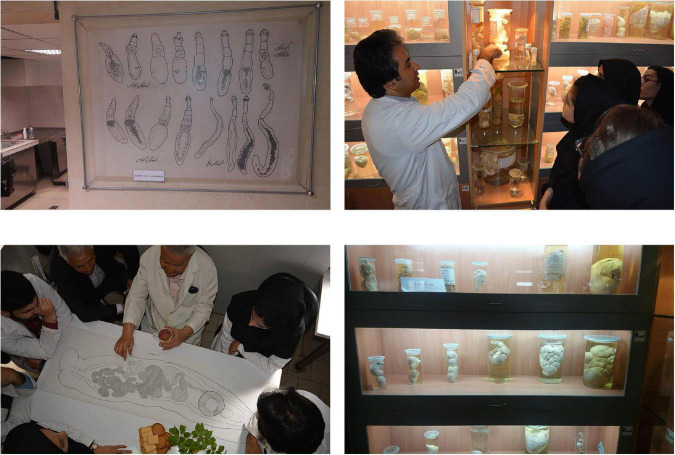
The role of the Iranian Parasitology Museum in science communication for the public and students. The figures were prepared from the archives of the Iranian Parasitology Museum.

The Iranian Parasitology Museum (IPM) provided an opportunity to explore practice in science communication among the general public and school children, while museum collections are freely available for translation of investigations by communications practitioners. The IPM is being answered questions about animal and human parasites (e.g., zoonotic parasites) and their life cycle, and infection consequences of parasites, as well as those regarding the controls required (Figure 3).

The success of preventive measures requires appropriate communication approach (such as supportive dialogue and participation) and proper interaction with policy makers under effective synergistic collaboration (interdisciplinary and interinstitutional cooperation) in the domain of OH approach. Establishment of an OH commission and/or OH joint steering committee at national level (54) can be capable of solving challenges and friction and facilitating relevant programs.

### Preventing illegal wildlife hunting and trade

Preventing illegal hunting of wildlife is also one of the ways to limit the spread of *Trichinella* sp. infection. Ecological consequences and public health zoonotic disease emergence from hunting waste, consumption, and use of wildlife are regarded as consequences of illegal wildlife hunting and trade.

Illegal wildlife trade (IWT) originates from lower levels of communities (e.g., poachers, local market sellers, and local consumers) especially in underdeveloped countries due to factors such as low socioeconomic status (e.g., low income, poverty and lower education), (55, 56).

The risk of zoonotic diseases spillover is associated with drivers such as land-use change (LUC), human culture and habits including hunting behavior, illegal wildlife hunting and trade for trophies and bushmeat), agriculture expansion, infrastructure development, urbanization and food industry change, as well as introduction of exotic species (57–61).

Despite existing regulations regarding the wildlife hunting or trade, policies restricting illicit activities would not be fully effective in many areas of the world, bringing greater public health concerns because of lacking monitoring of wildlife status and their products, indicating a serious “OH” issue.

Illegal importation of meat and its products have been related to trichinellosis in some European countries (55, 62, 63).

Although wildlife trade is prohibited in Iran (64), implementing effective enforcement of law against it is often difficult due to needs of manpower and major financial resources (65).

A number of mitigation actions and policies including enforcement of regulation at local, national and international settings (further monitoring of local restaurants and eateries by assessing the type of meat, continuous inspection of wildlife trade markets, increasing surveillance at ports and airports, etc.), and awareness-raising (Health communication) are among strict measures under the control of the sanitary authorities for combating such illegal activities and prevention of infectious disease transmission (55), Although hunting waste can also provide potential sources of infection for wild boars and other wildlife, this cannot be considerable in Iran and Turkey due to the religious restrictions. Of course, proper disposal of dead animals is also necessary for avoiding scavenging.

The wild boars are important sources of infection for a limited portion of human population in Iran and turkey including religious minorities consuming pork meat, and indigenous hunters involving in illegal hunting (16, 43).

## Concluding remarks

Though there are rare records of human infections caused by *Trichinella* sp. nematodes in Iran to date, the possibility of human infection exists among old male hunters (16, 42). High risk behavior such as hunter-based life styles, and recreational hunting can be the cause of trichinellosis (66). The transfer of infected meat from one region or even country to another can also contribute to local outbreaks (67).

Regarding population growth of wild boar in the recent years in Iran, they are considered as potential source for zoonotic diseases such *Trichinella* infection among high-risk individuals (43).

Possible consumption of wild boar meat after hunting by Iranian Armenians, and Zoroastrians (16, 20, 42), suggests a significant potential threat of trichinellosis; thus, health communication (i.e., educational plans) for hunters, farmers and consumers especially among populations living in remote areas can be fruitful in reducing or eliminating risk of exposure to these potential sources of *Trichinella*, especially in high-risk areas, where most people were unaware of the association of wild boar meat consumption and trichinellosis. Although hunting waste can also provide potential sources of infection for wild boars and other wildlife, this cannot be considerable in Iran due religious restrictions. Of course, disposal of dead animals is also necessary for avoiding scavenging.

The low seropositivity rate or near absence of human cases in Iran is mainly due to Muslim beliefs based on not consuming wild boar meat or pork meet, although lack of further studies may be involved in such results to some extent.

Previously most Iranian published papers had reported *Trichinella* larvae from wild animal isolates as *T. spiralis*, because the multiple species concept was in dispute at the time (29), when three new species were proposed in 1972 based on biological features including *T. nativa* and *T. nelsoni* (68) and *T. pseudospiralis* (69).

More structured field surveys are required to elucidate the natural host distribution of *Trichinella taxa* in sylvatic carnivore of Iran.

Overall, *Trichinella* sp. has been reported from wild life of the Caspian region, Isfahan, Ardabil, Khuzestan, Khorasan Razavi, and Bandar Abbas in the north, central, northwest, southwest, north-east and of south of Iran. Current data on the distribution of *Trichinella* taxa is fragmentary. Moreover, most of available data comes from case reports and biological data of the *Trichinella* taxa in wild animals of Iran is very limited, while no data are available on the distribution of *Trichinella* taxa in many parts of Iran; thus, more epidemiological studies with larva identification at the species level by molecular tests are still needed for covering the whole country to ascertain the prevalence of *Trichinella* sp. infection, mode of transmission, and population structure in wild animals of Iran. Experimental studies are needed to elucidate the infectivity and predilection sites of *T, britovi* genotypes circulating in Iran. In Turkey, only a few studies on *T. britovi* infection have been published since no data have been reported on human trichinellosis after 2004 and wild boars after 2011. We believe that *Trichinella* spp. present in wildlife should be explored further, as this would greatly aid our understanding of epidemiology and to implement control strategies.

## Author contributions

MB, ML, SF, and MFH conceived the study and designed the study. MB, ML, and SF performed the search of the literature. SS, HA, MB, XW, and MFH contributed in data collection. All authors wrote the manuscript and read and approved the submitted version.
